# Injurious Symptoms from the Inhalation of Ether Vapor

**Published:** 1847-07

**Authors:** 


*Injurious Symptoms arising from the Inhalation of Ether Vapor.*—Among the numerous cases recorded in the foreign journals, in which ether has been inhaled for the purpose of preventing pain in surgical operations, there are some in which injurious effects have been produced. The following is related by Mr. Richardson, in the London Medical Gazette, April 2:

F. P. presented herself, March 6, 1847, for the extraction of a molar tooth of the lower jaw. She is a servant in a family, and eighteen years of age; is of a florid complexion, well nourished, and plump in appearance. She showed some nervous trepidation, and on sitting down began to shed tears. She was affected at the time with enlarged tonsils, but as they did not obstruct the free passage of the air, I thought they would not be an objection to the inhalation. She was desirous of having the ether. She had no complaint of the lungs or of the heart, and expressed herself perfectly well except the tonsils. She took the ether freely, having heard of the benefit others derived from it. After inhaling one minute, the tube was removed from the mouth. The muscles being contracted and the mouth firmly shut, there was a difficulty in opening it. As it took a little time to adjust the key, sensation returned, and she felt the pain of the extraction in an unmitigated degree. After the operation she bent her body backwards and sank out of her chair; she gradually got the better, however, of the effects of the ether, and began to cry, and consciousness returned; but in a short time she again lapsed into a state of unconsciousness, with convulsive movements of the muscles; but she again got better and attempted to walk, but could not. The convulsions returned; there were spasmodic movements of the muscles of the left side of the face, the angle of the mouth was drawn upwards, the head was hot, the cheeks flushed, the vessels of the conjuntiva injected, the pupils dilated; there were convulsive movements of the limbs, opisthotonos, and intense pain of the head. I endeavored to refrigerate the head by means of towels soaked in cold water, which somewhat relieved the symptoms. I proposed to bleed her, but she strongly objected, as she had come against her mother's wish, and she was afraid of her censure. This state of things continued about half an hour; the refrigeration afforded but partial relief, the convulsions became stronger, the spasmodic twitchings of the muscles of the face more frequent and more violent, the pupil more and more dilated, the pulse full, hard and bounding. There was no stertor of the breathing, nor was the countenance livid; on the contrary, the cheeks resembled two pieces of scarlet velvet; the breathing was not hurried.

I now found that bleeding was indispensable, and she consented; I therefore opened a vein. The blood did not flow very freely, but I took a full bleeding (eighteen ounces) from the arm, with marked relief to the symptoms. The convulsions ceased; convulsions which only returned at intervals previously now became established. At the end of the bleeding the pupil contracted on exposure to light; still there was spasmodic twitching of the muscles of the left side of the face. I directed the cold cloths to the head to be continued when the muscular twitchings went off, and she began laughing. She then sat up and arranged her hair, which had become disheveled during her struggles. She declared that she was quite free from headache, and her head felt quite well except a little confusion. She got up and walked home, a distance of about two hundred yards.

The following was the report of a person I requested to see her in the evening: "She is excited, but not more so than might be expected to be found in a nervous and hysterical patient. Headache, and pulse of eighty, neither particularly full nor throbbing; pupil active, eye heavy and tearful; catamenia present since yesterday." Ordered evaporating lotion to the head, five grains of calomel at bedtime, and a black draught.—In the morning I saw her again.

On the 19th she had been leeched for headache; pulse seventy-six, soft. There has been no return of convulsions since the bleeding from the arm. She feels giddy upon sitting up, and the auditory nerves are acutely sensitive to sounds.

Having entertained a very favorable opinion of the inhalation of ether from the many successive instances I had witnessed, I must say this and other cases have entirely altered my opinion, and very reluctantly am I induced to look upon the remedy as unsafe.

---

The same journal for April 9th contains the following case, related by J. W. Eastman, Esq.:

Albin Burfitt, of Silton, aged eleven years, became entangled, about 8 o'clock, a. m., on the 23d of February, in the machinery of a mill, in consequence of which he sustained a very severe compound fracture of the left thigh, with great laceration of the soft parts, and a simple fracture of the right thigh. Mr. Newman, of Mere, a very able and experienced surgeon, saw the patient soon after the accident, and, with the concurrence of another surgeon, Mr. Rumsey, determined on amputating the limb as soon as the patient had recovered from the shock of the accident. I was requested to be present at the operation.

Saw the case at 4 o'clock, p. m., and instantly agreed in opinion, as to the necessity of an operation, with the other surgeons, who had been in constant attendance on the boy throughout the day. We did not at this time entertain any fears of the patient's death, either from the injuries sustained or the intended operation. The nervous system had certainly received a great shock; but as there had been but a trifling loss of blood—as considerable reaction had been established—as no vital organ had apparently been injured—as the boy was quite sensible, and had shown great fortitude and patience under his severe sufferings—we all considered there was sufficient vital force in his system to support him under the operation.

The question now arose, whether the inhalation of ether should be employed. After mature consideration, we determined it should, and that we would use the apparatus we had at hand. As soon as the patient was brought under its influence, which was the case in about three or four minutes, Mr. Newman operated with his usual skill and judgement; but the patient's suffering on making the circular incision were so severe that the intelligent and humane clergyman of the parish, Mr. Martin, who personally waited on the boy throughout the operation, remarked that the remedy was quite a failure.

The inhalation was now employed the second time for two or three minutes, and with decided benefit as far as the entire suspension of suffering was involved, and the operation, in which the loss of blood was most trifling, was concluded. With its conclusion our difficulties and anxiety commenced, for our patient was in such a state of exhaustion and apparent intoxicatiou that we soon considered his life to be in danger; and our fears were but too fully realized, for in defiance of all the watchful attention it was in our united power to pay, he sank in less than three hours after the operation. The state of the brain during this period was peculiarly distressing: there were alternate manifestations of excitement and depression of the sensorial powers—at one time resembling delirium, at another like approaching syncope, and again like violent intoxication; and these alternate conditions continued until the poor boy died.

On a review of the above case, I am not only induced to consider that the inhalation of ether partially failed in suspending pain, but I also attribute the death of the patient to its narcotizing effects on the nervous system, previously depressed by great and extensive injuries.—*Med. News.*

